# Community Sewage Sensors towards Evaluation of Drug Use Trends: Detection of Cocaine in Wastewater with DNA-Directed Immobilization Aptamer Sensors

**DOI:** 10.1038/srep21024

**Published:** 2016-02-15

**Authors:** Zhugen Yang, Erika Castrignanò, Pedro Estrela, Christopher G. Frost, Barbara Kasprzyk-Hordern

**Affiliations:** 1 Department of Chemistry, University of Bath, Claverton Down, Bath BA2 7AY, UK; 2 Department of Electronic and Electrical Engineering, University of Bath, Claverton Down, Bath BA2 7AY, UK

## Abstract

Illicit drug use has a global concern and effective monitoring and interventions are highly required to combat drug abuse. Wastewater-based epidemiology (WBE) is an innovative and cost-effective approach to evaluate community-wide drug use trends, compared to traditional population surveys. Here we report for the first time, a novel quantitative community sewage sensor (namely DNA-directed immobilization of aptamer sensors, DDIAS) for rapid and cost-effective estimation of cocaine use trends via WBE. Thiolated single-stranded DNA (ssDNA) probe was hybridized with aptamer ssDNA in solution, followed by co-immobilization with 6-mercapto-hexane onto the gold electrodes to control the surface density to effectively bind with cocaine. DDIAS was optimized to detect cocaine at as low as 10 nM with a dynamic range from 10 nM to 5 μM, which were further employed for the quantification of cocaine in wastewater samples collected from a wastewater treatment plant in seven consecutive days. The concentration pattern of the sampling week is comparable with that from mass spectrometry. Our results demonstrate that the developed DDIAS can be used as community sewage sensors for rapid and cost-effective evaluation of drug use trends, and potentially implemented as a powerful tool for on-site and real-time monitoring of wastewater by un-skilled personnel.

Wastewater-based epidemiology (WBE) has been shown to be an innovative and promising approach for a cost-effective temporal evaluation of drug use trends^1^,^2^,^3^,^4^,^5^,^6^,^7^,^8^,^9^, compared to the conventional population surveys method. The drug consumption is evaluated by quantification of drug residues and/or metabolites in wastewater that enter the urban sewer system from urine and feces^6^,^10^. In the past decade, a variety of chemicals such as illicit drugs, prescribed pharmaceuticals and new pharmacologically active substances, have been analyzed in wastewater collected from wastewater treatment plants with the aim to evaluate community-wide use trends such as illicit drugs, tobacco and alcohol use^3^,^4^,^7^. The spatial difference and temporal drug use changes have been evaluated across Europe via the measurement of specific markers in wastewater from 42 cities in 21 European countries (total population 24.74 million)^5^. The analytical tools for drug use trends utilize mass spectrometry-based techniques due to their robustness, sensitivity and selectivity. The chemical compounds or their metabolites, so-called biomarkers, present in wastewater can be reliably quantified using an internal reference such as a deuterated analogue of target analyte and low-resolution mass spectrometry (e.g. triple quadrupole instruments). Non-targeted analysis is usually undertaken with the usage of high resolution mass spectrometry^10^. However, troublesome sample purification, costly measurements and the requirement for well-trained personnel may burden the assessment process. To this end, there is a great need for novel analytical tools to perform rapid and on-site analysis of wastewater with minimal sample processing by un-skilled personnel. Furthermore, the rapid monitoring with community sensors may minimize the uncertainty of WBE resulting from low stability of certain markers^6^,^11^.

Biosensing has emerged as a novel and powerful analytical technology in the fields of food safety, drug discovery, healthcare and environmental monitoring^12^. A biosensor is a small device with a biological receptor (peptide, DNA, protein, aptamer, etc.) that generates a physical signal such as optical, mass sensitive, electrochemical or mechanical signal in the presence of an analytical target. Electrochemical biosensors, in particular, provide a rapid response time, low cost and ability to be miniaturized with other portable devices for the on-site measurement requiring minimal sample processing even by un-skilled personnel. It is hypothesized that community sensors will play a crucial role in *in situ* quantitative analysis of chemicals, biomarkers and pathogens in wastewater, for the purpose of evaluation of community-wide drug use trends and monitoring of public health^13^. More importantly, community sewage sensors could provide real-time and continuous data for the government agencies in the monitoring of drug use trends, and may serve as an early warning sensing system for the agencies to make effective interventions. In particular, electrochemical sensors have shown a great promise in the detection of wastewater biomarkers such as mitochondrial DNA^14^. For instance, we recently developed a new label-free electrochemical DNA (E-DNA) biosensor utilizing a custom synthesized ferrocenyl intercalator as a transducer, which allows the detection of cancer associated human-specific mitochondrial DNA in wastewater and the identification of potential population biomarkers for monitoring of public health^14^.

Cocaine abuse is regarded as a global challenge, and the concentration in wastewater has been found to be in the range of 0.1–1 μg/L (0.3–3 nM)^5^,^7^. It is highly desirable to develop simple, rapid and cost-effective tools for the sensitive detection of cocaine. Sensors could play a vital role in monitoring cocaine abuse due to several advantages over conventional mass spectrometry-based analytical techniques. Aptamer sensors (aptasensors) have been employed, due to their low cost and simple design, as a new biosensing platform for the detection of cocaine in different matrices in the past decade^15^,^16^,^17^. An aptamer is an artificial single-stranded DNA or RNA oligonucleotide that is able to bind a specific molecular target with high affinity. Zhang *et al.*^18^ has developed a label-free impedimetric aptasensor on the basis of super-molecular aptamer fragments, capable to detect cocaine concentrations as low as 0.1 μM with a dynamic range from 0.1 μM to 20 μM. In this sensing platform, the recognition of cocaine by aptamer-modified electrodes changes the electron transfer resistance associated with the cocaine concentration. Cocaine in undiluted human saliva samples was also detected with a compact, integrated optofluidic system with combination of multiphase liquid-liquid extraction methods^19^. The limit of detection of the system reached 100 μg/mL (330 μM), but the sensitivity needs to be improved for detection of real cocaine in saliva samples which are at the μg/mL range^20^. Du *et al.*^21^ developed a label-free electrochemical aptasensor introducing a probe immobilization technique by the use of a layer-by-layer self-assembled multilayer with ferrocene-appended poly(ethyleneimine) on an indium tin oxide (ITO) array electrode for detection of cocaine. This electrochemical aptasensor can detect cocaine levels as low as 0.1 μM with a dynamic range from 0.1 μM to 38.8 μM. However, the challenges of aptasensors remain on the effective immobilization of ssDNA probe on the electrode surface and prevention from the formation of secondary or tertiary DNA structures and fouling onto the electrode surface.

In this paper, we present a novel approach to effectively immobilize aptamer on a gold electrode surface for the electrochemical detection of cocaine in wastewater. A DNA-directed immobilization aptamer sensor (DDIAS) was further implemented for the detection of small molecules such as cocaine on the basis of previously established immobilization strategy for the detection of proteins^22^. The surface density was optimized to allow DDIAS for the detection of cocaine concentrations as low as 10 nM, spanning a wide dynamic range from 10 nM to 5 μM. The matrix effects on the aptasensor were determined, and DDIAS was used for the detection of cocaine in wastewater prior to a simple pre-concentration step. The evaluation of cocaine occurrence concentration in wastewater demonstrated that the drug use during weekend was larger than that on working days, and the results were comparable with mass spectrometry data. To the best of our knowledge, it is the first time an aptasensor has been reported for the analysis of cocaine in wastewater with an aim of the evaluation of community-wide cocaine use trends via WBE.

## Results and Discussion

### DDIAS for COC detection

The DNA-directed immobilization (DDI) has been shown to be a promising strategy for the elaboration of biosensors to detect protein and antigen^23^,^24^,^25^. Typically, a protein/carbohydrate molecule tailed with complementary DNA is assembled onto the surface of solid support by hybridization with ssDNA probe, and the assembled protein/carbohydrate could be specifically recognized with target such as antigen/protein. However, the introduction of probes onto the DNA tail requires complicated synthesis and purification steps, and is likely to influence the affinity of the probe molecules. In our recent report^22^, we implemented the strategy using an aptamer to replace the DNA tailed probe for the specific detection of prostate specific antigen (PSA), and in-solution method within DDIAS is able to detect PSA as low as fM range due to a favorable binding medium (in a homogeneous solution). Compared to typical direct immobilization of aptamer onto electrodes, DDIAS simplified the elaboration of the biosensor and the linear linkage of DNA allows sufficient access for the active site of the aptamer to recognize the target^22^. Furthermore, a filtration device was employed to remove the unbound aptamer in solution pre-assemble onto chips to minimize the spurious effects. However, for the detection of cocaine, the filtration device could not be employed to separate the aptamer and aptamer/cocaine complex due to the low molecular weight of cocaine (Mw = 303.3 g/mol) and only small differences between aptamer and aptamer/cocaine complex. The in-solution approach of DDIAS therefore may not be a suitable method for cocaine detection as the “free” aptamer in solution may introduce a false positive response.

**Figure 1:**
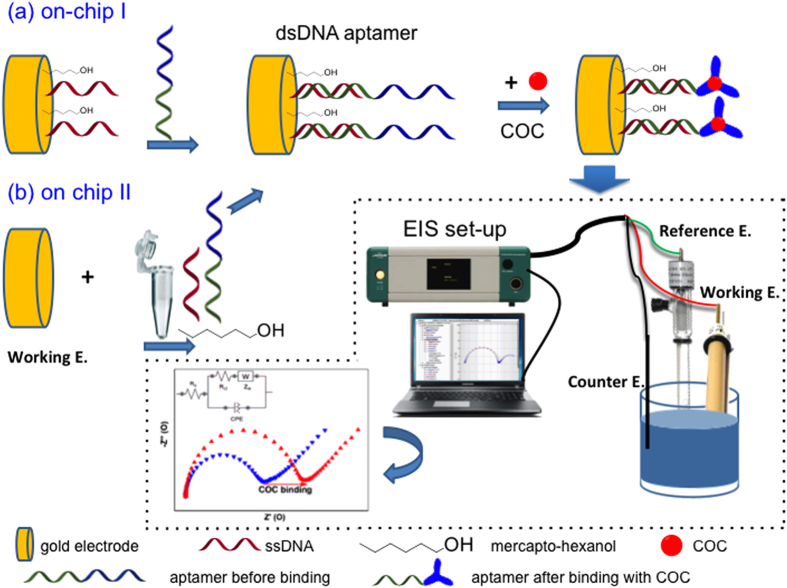
Schematic illustration of the DDIAS for detection of cocaine within ‘on-chip I’ (**a**) and ‘on-chip II’ (**b**) approach using EIS.

In this paper, we aimed to implement on-chip approach within DDIAS for the specific detection of small molecules such as cocaine, in order to demonstrate a generic sensing immobilization strategy that can be applied to monitor other biomarkers within WBE. The DDIAS on-chip I and II approach for the detection of cocaine is illustrated in Fig. 1, and the detail for the immobilization was described in the Methods section.

**Figure 2:**
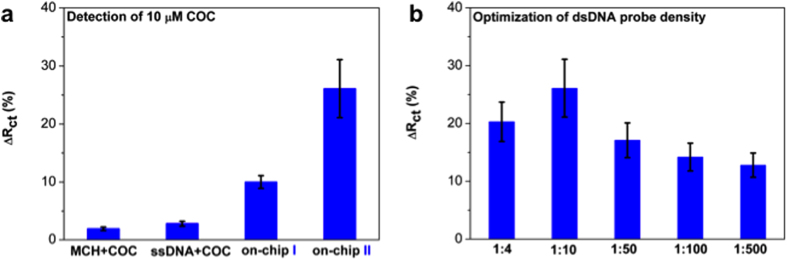
Comparison of different methods of DDIAS for the detection of cocaine (**a**) and optimization of surface density by changing the ratio between dsDNA probe and MCH using on-chip II approach within DDIAS (**b**); Each measurement was repeated at least on three independent electrodes to determine the standard deviation.

Figure 2 shows the shift of charge transfer resistance (Δ*R*_ct_) of the DDIAS for the detection of 10 μM cocaine with “on-chip I” and “on-chip II” approach. The binding of cocaine to the aptamers causes a change in overall charge distribution on the electrode surface, which in turn affects the electrostatic barrier that the negatively charged redox couple in solution needs to overcome in order to exchange an electron with the electrode – *i.e.* there is a change in the measured charge transfer resistance (*R*_ct_) of the system. For DDIAS, cocaine binding with aptamer either with “on-chip I” or “on-chip II” approach increased the charge transfer resistance increased by 10.0% and 26.1%, respectively. During electrochemical impedance spectroscopy (EIS) measurements, negatively charged ferri/ferrocyanide couple [Fe(CN)_6_]^3−/4−^ was utilized as redox marker to electrochemically characterize the signal induced by the conformation change of aptamer before and after binding with cocaine, thus the diffusion of [Fe(CN)_6_]^3−/4−^ and mass loading of cocaine onto the electrode surface play a significant role in the Δ*R*_ct_ value. Before binding with cocaine, the DNA aptamer adopts a so-called two-stem loop; however, upon cocaine binding it forms a further loop entrapping the cocaine molecule (so-called three-way junction^17^,^26^ (see illustration in Fig. 1). Therefore, the *R*_ct_ shifts to a high value upon the formation of three-way junction. For the on-chip II method, dsDNA was co-immobilized with MCH and it induced a bigger Δ*R*_ct_ value than on-chip I method, indicating that on-chip II approach is a promising immobilization strategy for cocaine detection. This was associated with the fact that the dsDNA aptamer probes within on-chip II has less steric hindrance for cocaine to be pocketed and thus allow more available aptamer to be exposed to bind with cocaine molecules on the electrode surface. Besides, it is likely that the dsDNA may prevent the two-stem loop structure of DNA aptamer fouling onto the electrode surface. Consequently, the on-chip II approach has a great promise for cocaine detection.

Two controls consisting of a MCH layer without DNA and a ssDNA probe layer without aptamer were used against 10 μM of cocaine. As shown in Fig. 2A, the response for these control electrodes is very weak or negligible indicating that ssDNA cannot specifically bind with cocaine and the electrode surface functionalized with MCH or MCH/ssDNA could limit the non-specific adsorption. The mass loading of cocaine onto MCH and MCH/ssDNA layers slightly increases Δ*R*_ct_, due to the hindrance of redox marker approaching onto the electrode surface. The electrodes modified with ssDNA probe are negatively charged, and the redox maker diffusion to the electrode surface is hindered due to repulsive interactions.

The molar ratio between dsDNA probe and MCH on electrodes is crucial for the DDIAS, as it determines the surface density of available aptamer to recognize cocaine. Additionally, co-immobilization of MCH serves as limiting non-specific adsorption, which is vital for the detection in complex samples. Keighley *et al.*^27^ optimized the ssDNA probe surface density by changing the ratio between ssDNA and MCH and obtained an optimal ssDNA mole fraction at 20% (ssDNA:MCH 1:4) for the highest Δ*R*_ct_ using EIS measurement. The effect of molar ratio between dsDNA probe and MCH on on-chip II approach within DDIAS was also investigated for sensitive cocaine detection. As shown in Fig. 2B, the Δ*R*_ct_ value firstly increased then slightly decreased as the increasing molar fraction of MCH, and the ratio of 1:10 (ssDNA:MCH) provided the highest response. The surface densities of ssDNA and dsDNA with the optimized molar fraction are in the low range, which enables efficient attachment of the target molecule as demonstrated by many studies^27^,^28^,^29^. However, the optimized molar fraction of dsDNA (1:10) aptamer probe for targeting cocaine shows an even lower probe density than that of ssDNA (1:4) for hybridization with complementary DNA. This is associated with the fact that the conformation of aptamer will change to the three-way junction form after binding with cocaine, which may need more available spaces on the surface.

However, as the ratio of dsDNA/MCH goes down to less than 1:10, it will reduce the available aptamer to bind with cocaine. In fact, the ratio of ssDNA probe and MCH was also optimized to 1:10 for a PSA assay using in-solution approach within DDIAS in our earlier report^22^, due to protein requiring a large space to attach the probe on the surface. The result therefore agrees well with our early report on impedimetric detection of DNA^27^,^30^ and protein^22^ by co-immobilization of DNA probe with MCH.

### Analytical performance of DDIAS

**Figure 3:**
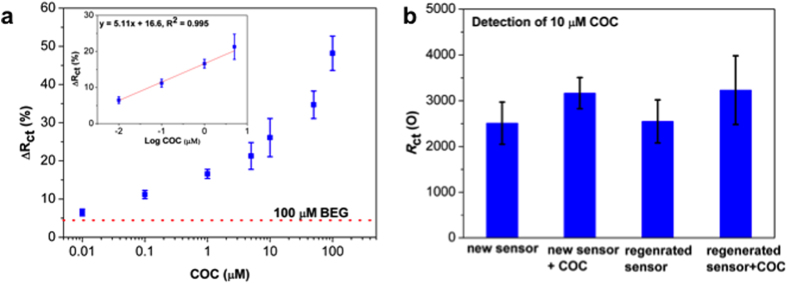
Δ*R*_ct_ from the detection of various concentrations of cocaine and the dynamic range of the dose response (insert) (**a**) and regeneration of DDIAS (**b**).

As shown in Fig. 3, the dose responses of DDIAS for cocaine and the non-specific adsorption to its metabolite benzoylegonine (BEG) were evaluated. Under the optimized conditions, DDIAS was employed to detect various concentrations of cocaine for evaluation of limit of detection (LOD) and dynamic range. As shown in Fig. 3A, Δ*R*_ct_ values increased with increasing cocaine concentration, and DDIAS allow for the detection of cocaine concentrations as small as 10 nM (3.03 μg/L) with a Δ*R*_ct_ value at 6.5%, which is even higher than that (5.0%) from detection of 100 μM BEG as a control for evaluation of non-specific adsorption. The LOD was estimated to be around 0.01 μM (3.03 μg/L) (3σ) for the on-chip II approach within DDIAS, and a dynamic range spanning from 10 nM to 50 μM (R^2^ = 0.995). In the above mentioned Zhang and co-worker’s report^18^, LOD of cocaine sensor was determined as 0.1 μM with a dynamic range from 0.1 μM to 20 μM. Sheng *et al.*^31^ developed an ultrasensitive electrochemical impedimetric aptasensor using an artificial 3D triangular pyramid frustum DNA nanostructure, which allows for the nanostructure being “close” and “open” before and after binding with cocaine. This conformation changes induce significant shift of charge transfer resistance, and LOD at 0.21 nM was achieved with a dynamic range spanning from 1 nM to 2.0 μM. However, the process of assemble and de-assemble 3D DNA nanostructures is difficult to control and the regeneration was not evaluated. Compared to other aptasensors based on the 2D conformation change upon binding with cocaine, our DDIAS is able to achieve a sensitive detection of cocaine without labeling and signal amplification. More importantly, the immobilization strategy was robust and DDIAS allows the re-usability of the sensors.

The stability of the DDIAS was also evaluated by measuring *R*_ct_ before and after the incubation of electrodes with 5 M sodium chloride (NaCl) solution, and the response (data not shown) has little change from interference of concentrated NaCl solution. This indicates that developed cocaine aptasensor within DDIAS using on-chip II method is also able to withstand salt solutions and will be beneficial for the detection of cocaine in various samples such as wastewater, which has the same profile of DDIAS using in-solution approach for detection of PSA^22^.

**Figure 4:**
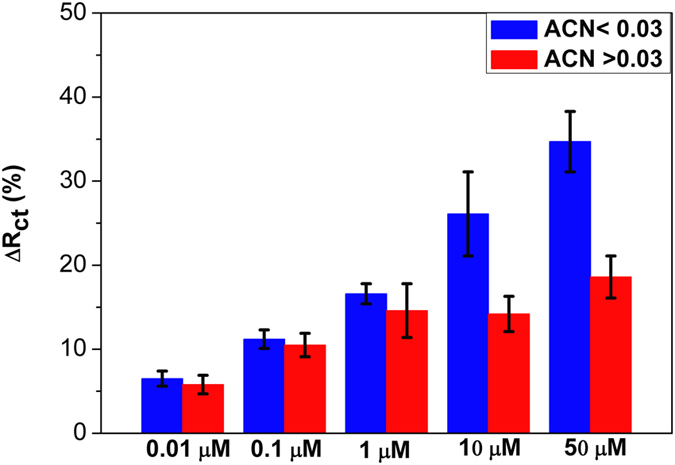
Effects of solvent on the binding efficiency between cocaine and the aptamer.

To further evaluate our developed aptasensor, a batch of 8 electrodes after immobilization of dsDNA probes was used to detect cocaine (10 μM) and followed by heating the sensor chips in ultrapure water/methanol (v/v 4/6) mixed solution at 60 °C for 1 h. The heating step unfolds the aptamer and releases the captured cocaine molecules^18^. The sensor chips were then re-used to detect cocaine. As shown in Fig. 4A, the *R*_ct_ value from regenerated chips before and after binding with cocaine has negligible changes, indicating that our DDIAS can be regenerated with a simple temperature step.

We also found that the effect of solvent has an effect on the binding efficiency between aptamer and cocaine. Acetonitrile (ACN) is a widely used stock solvent for commercialized cocaine products for research purposes. Kang *et al.*^32^ have demonstrated that the association constant strongly depends on the percentage of ACN in the cocaine solution. Typically, the percentage of ACN at 0.03 is a threshold value for the affinity between cocaine and anti-cocaine aptamer, with reduced affinity above this value. In our DDIAS, the Δ*R*_ct_ value also depends on the percentage of ACN in the binding buffer. In the lower cocaine concentration range within ACN percentage less than 0.03, Δ*R*_ct_ value was proportional to the cocaine dose (Fig. 4, blue bars). However, for higher ACN concentrations, the Δ*R*_ct_ value saturates for cocaine concentrations above 1 μM (red bars). These results are in good agreement with the affinity study described in reference^32^, and this should be taken into account in the development of cocaine aptasensor. To avoid the effect from solvent on our DDIAS, we used the initial concentration of cocaine at 1 mg/mL for gradual dilution and to make sure that the ACN percentage was less than 0.03 in all samples, and a typical dose-response Δ*R*_ct_ value was presented in Fig. 4 (blue bar).

### Quantification of cocaine in wastewater for evaluation of drug consumption

**Figure 5:**
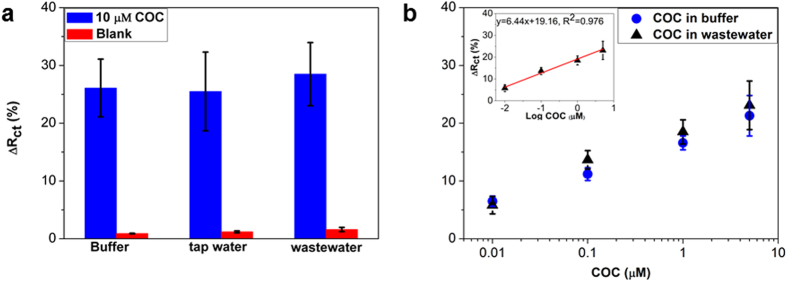
Detection of 10 μM cocaine in different matrix (buffer, tap water and wastewater) using DDIAS (**a**) and comparison of the response of the spiked cocaine in buffer and wastewater (**b**).

Figure 5A shows the matrix effects on the spiked cocaine in buffer, tap water and wastewater. Before spiking, wastewater was treated with a 10 K filtration device in order to remove the larger biomolecules, which may have an effect on the response from the blank. The response from the wastewater was negligible, indicating good anti-fouling properties of the sensor. The Δ*R*_ct_ value therefore did not show noticeable differences from cocaine spiked to the three matrices, indicating that our DDIAS is selective for cocaine detection. A series of cocaine concentrations within the range 10 nM to 5 μM were also spiked to wastewater, and the results show the response to be quite close to that from cocaine in standard buffer (Fig. 5B). This suggests that our DDIAS is promising to limit the non-specific adsorption and the high affinity of aptamer could selectively recognize cocaine in wastewater.

**Figure 6:**
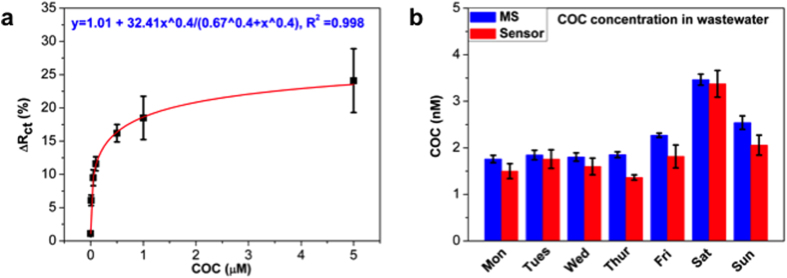
Calibration curve of cocaine in wastewater using a non-linear fit (Hill 1 for dose growth) (**a**) and quantification of cocaine concentration in wastewater collected from seven consecutive days within a week using developed DDIAS and mass spectrometry (**b**).

The optimized DDIAS was used to detect cocaine in wastewater collected daily during one week monitoring, with an aim to quantify cocaine for estimation of drug consumption at the community level. The cocaine concentration in wastewater was estimated to be within the nM range^5^,^7^, which is below the LOD of our DDIAS. Therefore, wastewater was pre-concentrated 100 times using solid phase extraction (SPE) in order to allow for the quantification of cocaine using the aptasensor. The calibration curve (Fig. 6A) was obtained by spiking various concentration of cocaine into a wastewater sample within the linear range. The cocaine concentrations in real daily wastewater samples were quantified using DDIAS based on the calibration curve, which is a typical dose response of the Hill type (*y* = *y*_0_ + *y*_1_*c*^*n*^/(*k*^n^ + *c*^*n*^) (where *c* is the concentration, *y* is the measured signal, *y*_0_, *y*_1_, *k*, and *n* are the parameters for fitting) and provides better fitting results than a linear fit (data not shown). The Hill equation assumes a sigmoidal shape that can be approximated well by means of the 4-parameter non-linear logistic equation, which is commonly employed to fit biological dose response curves^33^. As shown in Fig. 6B, the cocaine concentration in wastewater depends on the day of sample collection, and it is generally higher on the weekend than that on the week days. The cocaine consumption during the weekend is usually higher than those during working days from most of European cities^5^,^7^. However, it is essential to take into account other parameters such as rainfall and variable flow of wastewater (that can affect concentrations of cocaine in wastewater) for the estimation of the real cocaine daily load. Cocaine daily load estimated by DDIAS and flow rate of wastewater are presented in Table 1.

In order to validate the results and estimate the cocaine consumption in a defined population, a chromatography assay coupled with tandem triple quadrupole mass spectrometry was performed to quantify the concentration of cocaine in wastewater. As shown in Fig. 6B, the quantified cocaine concentration is in good agreement with the concentration occurrence pattern determined from DDAIS. Although a pre-concentration (1 to 100) step was performed to meet the LOD and limit of quantification (10 σ) of designed DDIAS, the evaluation of drug occurrence concentration by DDIAS is more rapid and cost-effective, compared to conventional mass spectrometry technique. Furthermore, we hope to develop a simple portable pre-concentration device for on-site detection of wastewater coupled with an integrated fast and selective sensor assay, which may enable the quantification of the cocaine concentration pattern in real-time. As discussed above, the quantitative mass spectrometry analysis confirmed high selectivity, accuracy and precision of the developed sensor and proved that such sensors can be used for the rapid estimation of the community-wide drug consumption. We believe that this rapidly acquired data could also be vital for estimation of a threshold value for the preliminary indication of cocaine abuse in a defined community, which may be of great help for the government agencies to make effective interventions for illicit drug abuse. As a result, we demonstrated that an aptasensor could be used for cost-effective and fast estimation of community-wide drug use trends using WBE, and with a strong potential to be miniaturized for a portable assay at the site of sample collection.

## Conclusion

A novel aptasensor was developed on the basis of DDIAS for the detection of cocaine in wastewater to evaluate the illicit drug use trend via WBE. Two methods of DDIAS were compared and on-chip II approach showed to be more promising for the detection of cocaine. The surface density of aptamer probe was optimized by co-immobilization of MCH with dsDNA for an efficient binding with cocaine. The optimized DDIAS allows for the detection of cocaine at levels as low as 10 nM, which is highly sensitive when compared to other reported cocaine sensors based on signal transducer with aptamer conformation changes. We used the optimized DDIAS to detect cocaine in wastewater collected during 7 consecutive days, and quantification of the cocaine concentration pattern shows that it has higher cocaine consumption during the weekend than that on weekdays. The results are in good agreement with those analyzed by means of mass spectrometry. In consequence, we demonstrated that our developed DDIAS could be employed as an innovative analytical tool (named “community sewage sensor”)^13^ for the evaluation of community-wide drug use trends within WBE. We believe that community sewage sensors will open a new direction in monitoring of drug use trends and public health such as obesity, cancer and cardiovascular disease.

## Methods

### Materials

Gold disc working electrodes with a radius of 1.0 mm were purchased from IJ Cambria Scientific Ltd (Cambridge, U.K.). Oligonucleotides such as probe sequence 5′- TCA CAG ATG ACT-3′ and aptamer against cocaine: 5′- GAC AAG GAT AAA TCC TTC AAT GAA GTG GGT C ACT CAT CTG TGA -3′ were custom synthesized from Sigma-Aldrich (Gillingham, U.K.). DNA aptamer against cocaine was employed for the detection of cocaine elsewhere^16^,^17^,^34^. ssDNA probe (5′- TCA CAG ATG AGT-3′) was modified with a thiol group on the 5′ end to obtain HS-(CH_2_)_6_–ssDNA to assemble on gold electrodes. The HPLC grade DNA powders were aliquoted in 10 mM Tris-HCl and 1mM ethylenediaminetetraacetic acid (EDTA) solution upon receipt and stored at −20 °C for long-term usage. The reference standard of cocaine (Mw 303.35 g/mol, 1 mg/mL in acetonitrile) was purchased from LGC (Cerilliant, U.K.). Cocaine-D^3^ (M_w_ 306.37 g/mol) (LGC, Middlesex, U.K.) was the deuterated analogue of the target analyte used as an internal standard (IS). Both analytical standards were of high purity (100%) according to their certificate of analysis. Thiolated single-stranded DNA (ssDNA), 6-mercapto-hexanol (MCH), methanol, acetonitrile (ACN) and ammonium acetate bezoylecognine (BEG) and all other chemicals were purchased from Sigma-Aldrich and used as received unless otherwise stated. All the solvents were of HPLC grade. Ultrapure water was obtained from the PURELAB UHQ-PS Unit (Elga, U.K.)

The buffer for immobilization of ssDNA probe consisted of 0.8 M phosphate buffer (PB) with 1.0 M NaCl, 5 mM MgCl_2_ and 1 mM ethylenediamine tetraacetic acid (EDTA), pH 7.0. The DNA hybridization buffer and cocaine incubation buffer was composed of 50 mM PB with 100 mM K_2_SO_4_ (pH 7.4).

### Sample collection and preparation

The influent daily 24-hour composite raw wastewater was collected from a local wastewater treatment plant consecutively in a week from March 10^th^ to 16^th^, 2015. Wastewater was transported back to the laboratory in cool boxes packed with ice blocks and was processed on arrival or was frozen at −20 °C upon arrival and stored until analysis with the sensor.

The wastewater was filtrated and pre-concentrated with a solid phase extraction (SPE), as described elsewhere^35^. In brief, the wastewater samples (100 mL) were filtered and then spiked with cocaine-D3 (1 μg L^−1^) before being passed through Oasis HLB cartridges (Waters, U.K.). Oasis HLB cartridges (60 mg, Waters, UK) were used for performing solid phase extraction (SPE) of the samples according to the following protocol. The cartridges were conditioned with 2 mL methanol followed by equilibration with 2 mL ultrapure water at a rate of 3 mL min^−1^. 100 mL of composite wastewater sample spiked with IS were deposited into the HLB cartridge at a rate of 8 mL min^−1^. The cartridges were then washed with 3 mL of ultrapure water at a rate of 3 mL min^−1^. The elution was carried out with 4 mL methanol at a rate of 8 mL min^−1^ into 5 mL previously silanised glass tubes. The extract was transferred to the TurboVap evaporator (Caliper, UK) and dried under nitrogen flow (5–10 psi) at 40 °C. After reconstitution with 500 μL 1 mM ammonium acetate/methanol (v/v 85:15), the samples were filtered through 0.2 μm polytetrafluoroethylene (PTFE) filters (13 mm, Whatman Puradisc). The filtered samples were then transferred to polypropylene plastic vials bonded pre-slit PTFE/Silicone septa (Waters, UK) and 20 μL were directly injected into an ultra-performance liquid chromatography-tandem mass spectrometer (UHPLC-MS/MS) system.

The analysis of wastewater by DDIAS was performed with the same SPE protocol, except reconstituted with measurement buffer of EIS. The samples were analyzed immediately after SPE.

### Cleaning of electrodes

Gold electrodes were polished with 50 nm aluminium oxide particles (Buehler, Lake Bluff, Illinois, U.S.A.) on a polishing pad (Buehler, Illinois, U.S.A.) for 5 min, followed by sonication in ultrapure water to remove any particles. Electrodes were immersed in piranha solution (H_2_SO_4_/H_2_O_2_, v/v 7/3) for 1 min followed by rinsing thoroughly with deionized water, and ready for electrochemically cleaning. Electrodes were electrochemically cleaned in a classical three-electrode cell as described elsewhere^14^,^22^,^27^. In brief, electrodes were immersed in H_2_SO_4_ (0.5 M) solution and the potential was scanned between the oxidation and reduction potentials of gold, from 0 V and +1.5 V *versus* an Ag/AgCl reference electrode, with scanning rate at 0.2 V/s and step potential at 0.01 V/s for 60 cycles until there was no further change in the voltammogram.

### Elaboration of DDIAS for cocaine detection

The aptamers were synthesized with a tail of twelve nucleotides as a complementary sequence to the ssDNA probe. Two on-chip approaches of the so-called “DNA-directed immobilization aptamer sensor” (DDIAS)^22^ were designed for cocaine detection: “on-chip I” and “on-chip II”. In the ‘on-chip I’ method, electrodes were exposed to mixed ssDNA probe/MCH solution for 16 h in a humidity chamber at 4 °C. The molar ratio between ssDNA/dsDNA probe and MCH was 1:10 or otherwise stated and DNA probe was set at 1 μM in immobilization buffer. After ssDNA was immobilized on the gold electrodes, complementary DNA aptamer was hybridized with ssDNA probes on the electrode surface. In the “on-chip II” method, the complementary aptamer DNA was incubated with ssDNA in solution at equivalent molar ratio for 1 h; and the dsDNA probe was co-immobilized with MCH at designed ratio onto electrodes surface.

After immobilization, electrodes were rinsed in 50 mM PB with 100 mM K_2_SO_4_ and 10 mM EDTA (pH 7.0) to remove any remaining Mg^2+^. To ensure complete thiol coverage of the gold surface and make favorable DNA aptamer conformation for recognition of cocaine, electrodes were backfilled with MCH (1 mM, H_2_O) for 1 h, followed by rinsing with buffer and drying with nitrogen for measurement. The aptamer-probed electrodes were measured with EIS before and after 1 h incubation with various concentration of cocaine. For wastewater measurement, a centrifugal filtration device (Amicon Ultra-0.5 mL 10k device, Merck Millipore, U.K.) was used to treat the sample in order to reduce the background before spiking cocaine.

### Electrochemical impedimetric characterization of DDIAS

Electrochemical impedance spectroscopy (EIS) measurements were performed using a μAutoLabIII/FRA potentiostat (Metrohm, Schiedam, The Netherlands). A conventional 3-electrode configuration was used consisting of gold disc working electrode, a platinum wire counter electrode (ALS Instruments, Tokyo, Japan) and an Hg/Hg_2_SO_4_ (K_2_SO_4_ sat.) reference electrode (BASi, West Lafayette, IN, U.S.A.) placed into a salt bridge and against which all potentials are quoted. Before measurement, electrodes were placed into EIS buffer for 1 h to stabilize.

EIS was measured in a solution of 2 mM K_4_[Fe(CN)_6_]/2 mM K_3_[Fe(CN)_6_] in 50 mM phosphate buffer (PB) with 100 mM K_2_SO_4_ pH 7.0 (ionic strength 447 mM). The reference electrode was connected via a salt bridge filled with 50 mM PB with 100 mM K_2_SO_4_ (pH 7.0). The impedance spectrum was measured over the frequency range from 100 kHz to 1 Hz for 61 times, with a 10 mV a.c. voltage superimposed to a d.c. bias of −0.195 V, corresponding to the formal potential of the redox couple.

### Analysis of wastewater with mass spectrometry

All wastewater samples were analyzed using Waters ACQUITY UPLC® system (Waters, UK) and Xevo TQD Triple Quadrupole Mass Spectrometer (Waters, UK), equipped with an electrospray ionisation source in a positive ion mode. A CHIRALPAK® CBH HPLC Column 5 μm particle size, L × I.D. 10 cm × 2.0 mm (Chiral Technologies, Illkirch, France) with a Chiral-CBH guard column 10 × 2.0 mm, 5 μm particle size (Chiral Technologies, France) was used, and the column temperature was set at 25 °C. The mobile phase was composed of 1mM ammonium acetate/methanol (v/v 85:15) at a rate of 0.1 mL min^−1^ under isocratic conditions. ACQUITY UPLC^TM^ autosampler was kept at 4 °C. The injection volume was 20 μL. Nitrogen was used as the nebulizing gas, supplied by a high purity nitrogen generator (Peak Scientific, UK). Argon (99.999%) was the collision gas supplied by a BOC cylinder. MassLynx 4.1 (Waters, UK) was used to control the Waters ACQUITY system and the Xevo TQD. Data processing was carried out using the TargetLynx software (Waters, UK). Cocaine identification was assessed according to the European Guidelines concerning the performance of analytical methods and the interpretation of results.

The instrumental limit of detection (*IDL*) was determined at a concentration value giving a signal-to-noise ratio (S/N) ≥3 for all the MRM transitions selected for cocaine. The method detection limit (*MDL*) was calculated using the following formula:


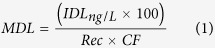


where *IDL* is the instrumental limit of detection, *R*_ec_ is the relative SPE recovery of the analyte in the matrix and *CF* is the SPE concentration factor.

The instrumental limit of quantification (*IQL*) was determined at the minimum concentration value giving S/N ≥ 10 for all the MRM transitions. The method quantification limit (*MDL*) was calculated using the following formula:


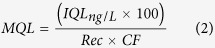


The linearity of the method was verified in the following range: *IDL* - 1000 μgL^−1^. The response was linear for cocaine.

Precision, expressed as relative standard deviation (RSD) of replicate analysis (*n* = 4) at three different concentrations on the same day (intra-RSD%), was evaluated as instrumental precision using standard solutions spiked in mobile phase whilst method precision using standard solutions spiked in influent wastewater.

Cocaine load that corresponds to the amount of cocaine arriving at the WWTP per day (g day^−1^) was determined according to our previously proposed approach^36^, which takes into account the stability of the analytes.

## Additional Information

**How to cite this article**: Yang, Z. *et al.* Community Sewage Sensors towards Evaluation of Drug Use Trends: Detection of Cocaine in Wastewater with DNA-Directed Immobilization Aptamer Sensors. *Sci. Rep.* **6**, 21024; doi: 10.1038/srep21024 (2016).

## Figures and Tables

**Table 1:** Concentrations and daily loads of cocaine in sewage determined with sensors and liquid chromatography coupled with tandem triple quadrupole mass spectrometry (LC-MS/MS) during the sampling period from March 10^th^ to 16^th^, 2015.

Date	Flow (m^3^/day)	C (ng/L) with sensors	Load (g/day) with sensors	C (ng/L) with MS	Load (g/day) with LC-MS/MS
Monday 16^th^	197493.3	455.3 ± 49.6	96.3 ± 15.8	533.5 ± 23.9	112.9 ± 7.1
Tuesday 10^th^	204490.8	533.5 ± 72.4	116.9 ± 18.5	560.0 ± 30.6	122.7 ± 9.5
Wednesday 11^th^	198950.4	485.2 ± 50.7	103.4 ± 15.9	546.5 ± 27.4	116.5 ± 8.3
Thursday 12^th^	197523	413.6 ± 49.4	87.5 ± 15.3	562.0 ± 18.6	118.9 ± 5.6
Friday 13^th^	252682.2	550.6 ± 98.9	149.1 ± 28.5	688.5 ± 14.0	186.4 ± 5.4
Saturday 14^th^	220687.2	1023.4 ± 99.2	242.1 ± 29.1	1051.0 ± 36.0	248.5 ± 12.0
Sunday 15^th^	193194	624.3 ± 82.6	129.2 ± 24.4	771.0 ± 44.5	159.6 ± 13.0
